# Investigating the contribution of hyaluronan to the breast tumour microenvironment using multiparametric MRI and MR elastography

**DOI:** 10.1002/1878-0261.13437

**Published:** 2023-05-03

**Authors:** Emma L. Reeves, Jin Li, Konstantinos Zormpas‐Petridis, Jessica K. R. Boult, James Sullivan, Craig Cummings, Barbara Blouw, David Kang, Ralph Sinkus, Jeffrey C. Bamber, Yann Jamin, Simon P. Robinson

**Affiliations:** ^1^ Division of Radiotherapy and Imaging The Institute of Cancer Research London UK; ^2^ Royal Marsden NHS Foundation Trust London UK; ^3^ Halozyme Therapeutics San Diego CA USA; ^4^ Division of Imaging Sciences and Biomedical Engineering King's College London UK; ^5^ Present address: Department of Circulation and Medical Imaging Norwegian University of Science and Technology Trondheim Norway; ^6^ Present address: Scintica Instrumentation Inc. London UK

**Keywords:** breast cancer, extracellular matrix, hyaluronan, multiparametric MRI, PEGPH20

## Abstract

Hyaluronan (HA) is a key component of the dense extracellular matrix in breast cancer, and its accumulation is associated with poor prognosis and metastasis. Pegvorhyaluronidase alfa (PEGPH20) enzymatically degrades HA and can enhance drug delivery and treatment response in preclinical tumour models. Clinical development of stromal‐targeted therapies would be accelerated by imaging biomarkers that inform on therapeutic efficacy *in vivo*. Here, PEGPH20 response was assessed by multiparametric magnetic resonance imaging (MRI) in three orthotopic breast tumour models. Treatment of 4T1/HAS3 tumours, the model with the highest HA accumulation, reduced T_1_ and T_2_ relaxation times and the apparent diffusion coefficient (ADC), and increased the magnetisation transfer ratio, consistent with lower tissue water content and collapse of the extracellular space. The transverse relaxation rate R_2_* increased, consistent with greater erythrocyte accessibility following vascular decompression. Treatment of MDA‐MB‐231 LM2‐4 tumours reduced ADC and dramatically increased tumour viscoelasticity measured by MR elastography. Correlation matrix analyses of data from all models identified ADC as having the strongest correlation with HA accumulation, suggesting that ADC is the most sensitive imaging biomarker of tumour response to PEGPH20.

## Graphical abstract

Diffusion‐weighted MRI and elastography can non‐invasively inform on preclinical breast tumour response to extracellular matrix modulation, specifically hyaluronan degradation by PEGPH20, and may facilitate the clinical development of stromal‐targeted therapies.

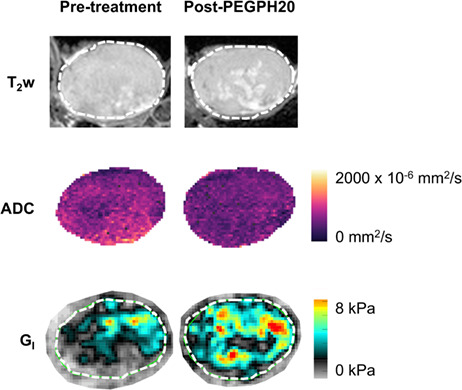

**Abbreviations**
ADC: apparent diffusion coefficient
CV_WS_
: within‐subject test–retest coefficient of variation
DAB: diaminobenzidine
DWI: diffusion‐weighted imaging
ECM: extracellular matrix
FBS: foetal bovine serum
FFPE: formalin‐fixed paraffin‐embedded
FOV: field of view
GAG: glycosaminoglycan
G_d_
: elastic modulus
G_l_
: viscous modulus
H&E: haematoxylin and eosin
HA: hyaluronan
HAS3: hyaluronan synthase 3
IFP: interstitial fluid pressure
IHC: immunohistochemistry
IS‐MRI: intrinsic susceptibility magnetic resonance imaging
MGE: multiple gradient echo
MRE: magnetic resonance elastography
MRI: magnetic resonance imaging
MTR: magnetisation transfer ratio
PEGPH20: pegvorhyaluronidase alfa
PTFE: polytetrafluoroethylene
R_2_*: transverse relaxation rate
RARE: rapid acquisition with refocused echoes
ROI: region of interest
SEM: standard error of the mean
SS: solid stress
STR: short tandem repeat
T_1_
: longitudinal relaxation time
T_2_
: transverse relaxation time
TE: echo time
TR: repetition time
TTP: total tissue pressure

## 1 Introduction

Malignant primary breast tumours are often characterised by a strong desmoplastic reaction, which results in a dense collagenous stroma with extracellular matrix (ECM) rigidity, providing a permissive scaffold for tumour growth. This stromal‐dense phenotype is associated with tumour progression (accelerated growth, invasion and metastasis) and poor drug delivery [1, 2]. Breast tumours with high metastatic propensity often have elevated expression of ECM components, including fibrillary collagens, laminins, fibronectin and hyaluronan, previously referred to as hyaluronic acid (HA) [3]. HA is a large, unbranched, negatively charged, hygroscopic glycosaminoglycan (GAG) consisting of repeating disaccharides of D‐glucuronic acid and *N*‐acetylglucosamine. HA is overexpressed in 25–30% of human tumours, including breast cancer in which the extent of HA accumulation is associated with metastasis and can predict for overall survival [4, 5, 6]. HA within the tumour microenvironment avidly binds a considerable number of water molecules (up to 15 per disaccharide unit), creating an immobile gel–fluid phase that induces swelling of the tumour ECM, elevated interstitial fluid pressure (IFP), collapse of local blood and lymphatic vessels, and tumour hypoxia [7, 8, 9].

Numerous stromal targeted treatment strategies and accompanying biomarkers are being developed to alter tumour–stroma interactions for therapeutic gain, including drugs targeted against HA [2, 10]. Pegvorhyaluronidase alfa (PEGPH20) is a PEGylated, recombinant, human hyaluronidase shown to enzymatically degrade HA, reduce IFP and decompress blood vessels, thereby enhancing drug delivery and treatment response to chemo‐, radio‐ and immunotherapy in a panel of stromal‐dense preclinical tumour models [11, 12, 13].

HA degradation (depolymerisation) by PEGPH20 has been associated with a decrease in tumour water content [7, 9]. This reorganisation of previously HA‐bound water molecules within the tumour microenvironment provides a strong rationale for using magnetic resonance imaging (MRI), which exploits the magnetic moment of protons in tissue water, to assess response to PEGPH20 *in vivo*. MR image acquisition can (a) be sensitised to numerous independent contrast mechanisms related to the physical state of tissue water and its ability to diffuse and (b) provide a direct measure of the viscoelastic properties of tissue (elastography). Parametric maps can be calculated and used to inform on tumour structure and function *in situ*.

In this preclinical study, we applied a multiparametric MRI strategy, incorporating the use of magnetic resonance elastography (MRE), to characterise orthotopic breast tumours derived from cell lines with differing levels of HA accumulation, and assess their subsequent response to PEGPH20 *in vivo* [14]. Our multiparametric MRI strategy focussed on quantitation of a panel of clinically translatable endogenous MRI biomarkers sensitive to different tumour tissue properties, specifically the native longitudinal (T_1_) and transverse (T_2_) relaxation times sensitive to the organisation of neighbouring water molecules (free, structured or bound), the magnetisation transfer ratio (MTR) which informs on the proportion of water bound to macromolecules, the apparent diffusion coefficient (ADC) sensitive to tissue water diffusivity, the transverse relaxation rate (R_2_*) sensitive to paramagnetic species including deoxyhaemoglobin in haemodynamic vasculature, and elastic (G_d_) and viscous (G_l_) components of the complex shear modulus that are measures of tumour viscoelastic properties. Pathological correlates from digital pathology of MRI‐aligned tissue sections were also sought. Collectively, the data were used to appraise these MRI biomarkers to (a) inform on HA accumulation in breast cancer models and their response to PEGPH20 and (b) evaluate whether the imaging biomarkers yield additional mechanistic insight into modulation of the ECM.

## 2 Materials and methods

### 2.1 Cell lines

All cell lines tested negative for *Mycoplasma* infection, and cell identity was authenticated by short tandem repeat (STR) profiling. luc‐MDA‐MB‐231 LM2‐4 highly malignant luciferase‐expressing triple‐negative breast cancer cells isolated from a lung metastasis were provided by Dr R. Kerbel (University of Toronto, Canada) and cultured in Dulbecco's modified eagle's medium (DMEM; Gibco, 31966, Paisley, Scotland) supplemented with 10% (v/v) foetal bovine serum (FBS; Pan Biotech, Wimborne, UK). 4T1 murine breast adenocarcinoma cells and 4T1/HAS3 murine breast adenocarcinoma cells which overexpress HA synthase 3 (HAS3) were provided under a research collaboration agreement with Halozyme Therapeutics (San Diego, CA, USA). 4T1 and 4T1/HAS3 cells were cultured in RPMI 1640 medium (Gibco, 72400) supplemented with 10% FBS with the addition of 100 μg·mL^−1^ hygromycin B (Gibco, 10687010) for HAS3 selection in 4T1/HAS3 cells.

### 2.2 Animals and tumour models

All animal experiments were approved by the Institute of Cancer Research Animal Welfare and Ethical Review Body, performed in accordance with the UK Home Office Animals (Scientific Procedures) Act 1986, the United Kingdom National Cancer Research Institute guidelines for the welfare of animals in cancer research, and reported according to the Animal Research: Reporting *In Vivo* Experiments (ARRIVE) guidelines [15, 16]. The project licences used for these experiments were PCC916B22 and PP3472375. Mice were adult (6–8 weeks old) and sourced from Charles River (Harlow, UK). Mice were housed in specific pathogen‐free rooms in autoclaved, aseptic microisolator cages with a maximum of 6 animals per cage. Mice were allowed access to food and water *ad libitum*.

Murine 4T1 and 4T1/HAS3 breast tumours were propagated orthotopically by injecting 1 × 10^5^ cells in 100 μL of sterile serum‐free medium into the third mammary fat pad of adult female BALB/c mice (4T1: one study *n* = 14; 4T1/HAS3: three studies total *n* = 24). Human luc‐MDA‐MB‐231 LM2‐4 breast tumours were propagated orthotopically in the third mammary fat pad of adult female athymic NCr‐*Foxn1* ^nu^ mice by injecting 2 × 10^6^ cells in 100 μL Matrigel (Fisher Scientific, Loughborough, UK) and serum‐free medium suspension (1 : 1 ratio; two studies total *n* = 26). Tumour development was monitored twice‐weekly using callipers. Tumour volumes reported herein were measured using multi‐slice anatomical T_2_‐weighted MRI. A total number of 64 mice were used for this research.

### 2.3 Formulation, administration and dosing of PEGPH20

PEGPH20, a PEGylated form of the recombinant human hyaluronidase PH20, was provided by Halozyme Therapeutics and used to enzymatically degrade tumour HA [7]. A 24‐h timepoint and single 1 mg·kg^−1^ dose were used [13]. PEGPH20 was diluted in saline and administered intravenously via a lateral tail vein. Control mice were treated intravenously with saline alone.

### 2.4 MRI data acquisition and analysis

Pre‐treatment MRI was performed when 4T1, 4T1/HAS3 and MDA‐MB‐231 LM2‐4 tumours reached 453 ± 23, 335 ± 23 and 472 ± 24 mm^3^, respectively (calculated using anatomical T_2_‐weighted MRI). Mice were given 5 h to recover from anaesthesia before administration of PEGPH20 or saline. MRI was then repeated 24 h later.

MRI data were acquired on a 7T BioSpec 70/20 USR horizontal MRI system (Bruker Instruments, Ettlingen, Germany) using a purpose‐built MRE platform, and a 4 cm volume coil positioned at the isocentre of the magnet [17].

For MRI, tumour‐bearing mice were anaesthetised with a 7 mL·kg^−1^ (4T1 and 4T1/HAS3) or 9 mL·kg^−1^ (MDA‐MB‐231 LM2‐4) intraperitoneal injection of Hypnorm™ (0.315 mg·mL^−1^ fentanyl citrate plus 10 mg·mL^−1^ fluanisone; Janssen Pharmaceuticals, High Wycombe, UK) and Hypnovel™ (5 mg·mL^−1^ midazolam; Roche, Welwyn Garden City, UK) and sterile water in a 1 : 1 : 2 ratio. Body temperature was maintained at approximately 37 °C using a water heating pad, and breathing rate was monitored using physiological monitoring equipment (SA Instruments, Stony Brook, New York, NY, USA).

Anatomical T_2_‐weighted images were first acquired using a rapid acquisition with refocused echoes (RARE) sequence (TE = 36 ms, TR = 4500 ms, RARE factor = 8, 20 contiguous 1 mm thick axial slices, matrix size 128 × 128 over a 3 × 3 cm field of view [FOV]) and used to determine the tumour volume, plan the subsequent functional MRI acquisition and optimise the local magnetic field (B0) homogeneity over the tumour using a localised map shim.

The subsequent multiparametric MRI protocol included the acquisition of inversion recovery (IR)‐TrueFISP images (TE = 1.7 ms, TR = 3.4 ms) for estimating the native T_1_ and T_2_ relaxation times (ms), MT‐RARE (TE = 19.22 ms, TR = 1500 ms, frequency offset = 25 ppm ‘on’ and 100 ppm ‘off’) to derive the MTR (%), respiratory‐gated diffusion‐weighted images (DWI; TE = 37.88 ms, TR = 1500 ms, 5 b‐values between 200 and 1000 s·mm^−2^) for determination of the ADC (×10^−6^ mm^2^·s^−1^), intrinsic susceptibility (IS‐MRI) using a multiple gradient echo (MGE) sequence (TE = 3 ms, TR = 200 ms, TE_SPACE_ = 3 ms, 8 echoes) to estimate R_2_* (s^−1^), and finally MRE (TE = 30 ms, TR = 504 ms, frequency = 1 kHz, amplitude = 10 V, 4 wave phases) to quantify G_d_ and G_l_ (kPa). The total acquisition time for all the sequences including MRE was ∼ 45 min.

The MRE set‐up was as previously described [18]. An external mechanical vibration was generated by an electromagnetic shaker (Brüel & Kjaer, Nærum, Denmark), transmitted through a flexible nylon rod to a square piston with a semi‐curved surface (1.2 × 1.2 cm, with various depth options between 2 and 7 mm). The piston was positioned in direct contact with the tumour. A cantilever allowed conversion of the horizontal vibration of the shaker into vertical vibrations onto the tissue.

Parametric maps of T_1_, T_2_, MTR, ADC and R_2_* were reconstructed using in‐house software written in IDL from a 1 mm thick axial slice over a 3 × 3 cm FOV taken from the tumour centre [19]. Parametric maps of G_d_ and G_l_ were reconstructed isotropically from three 0.3 mm thick axial slices over a 1.92 × 1.92 cm fov using software shared by Prof Ralph Sinkus. Median values for each tumour were calculated from a region of interest (ROI) which contained viable tumour tissue and excluded necrosis. Areas of necrosis were drawn on T_2_‐weighted images and confirmed by examination of aligned H&E‐stained histological sections.

### 2.5 Interstitial fluid pressure (IFP) measurement

Invasive tumour IFP measurements were performed *in vivo* immediately after post‐treatment MRI using a Millar Mikro‐Tip® piezoelectric mouse pressure catheter (model SPR‐1000, 0.33 mm diameter). To isolate IFP from total tissue pressure, the catheter was covered with modified polytetrafluoroethylene (PTFE) tubing (ID = 0.4 mm) [20]. The PTFE tubing was perforated to allow transmission of fluid pressure and minimise transmission of solid stress to the pressure sensor. Prior to each experiment, the catheter was placed in a water bath (∼ 37°C) until stabilisation and calibrated at 0 and 120 mmHg to a manometer. A 23‐gauge needle was introduced into and removed from the tumour tissue and then the covered catheter was quickly inserted into the pierced track. Care was taken to locate the pressure sensor at approximately the tumour centre. Data were collected for at least 5 min and average pressure after stabilisation was calculated using labchart (ADInstruments, Oxford, UK).

### 2.6 *Ex vivo* measurement of tumour water content

Guided by the post‐treatment T_2_‐weighted MRI, tumours were carefully dissected and bisected at the MRI plane. One tumour half was weighed, dried in a lyophiliser for 24 h and then reweighed. Tumour water content (%) was calculated using [7]:

$$\text{Water content}\;\left(\%\right)=\frac{\mathrm{wet}\;\text{weight}-\mathrm{dry}\;\text{weight}}{\mathrm{wet}\;\text{weight}\;}\times 100$$

### 2.7 Histology and immunohistochemistry (IHC)

The other tumour half was fixed in formalin and embedded in paraffin. Serial formalin‐fixed paraffin‐embedded (FFPE) tissue sections (5 μm) were cut using a microtome (RM2125RT, Leica, Milton Keynes, UK). Tinctorial haematoxylin and eosin (H&E) staining was performed for the assessment of nuclear density, morphology and necrosis. HA was detected by an affinity histochemistry assay using a biotinylated, recombinant HA‐binding protein (modified TSG‐6 probe HTI‐601, Halozyme Therapeutics) [21]. Collagen I & III were detected by picrosirius red staining (PRC/R/109, Pioneer Research Chemicals, Colchester, UK). Blood vessels were detected by CD31 immunohistochemistry using a rat anti‐mouse CD31 primary antibody (DIA‐310, Dianova, Hamburg, Germany) and Rat Histofine MAX PO (414311F, Nichirei Bioscience, Tokyo, Japan).

Whole‐slide images were digitised using a Nanozoomer XR scanner (Hamamatsu, Welwyn Garden City, UK). Nuclear density, HA (%) and blood vessel density were quantified at 40× magnification from H&E, HTI‐601 and CD31 staining, respectively, using definiens tissue studio® (Definiens, Carlsbad, CA, USA). The extent (%), fractal dimension and entropy associated with collagen I & III were quantified at 20× magnification from picrosirius red staining as previously described [18].

### 2.8 Generation of HA density parametric maps

HA density parametric maps were generated as a visual aid for the spatial comparison of MRI with histology within a single tumour and were not used for any numerical analyses. Whole‐slide images of HA staining were processed to match the MRI resolution (234 × 234 μm). The brown diaminobenzidine (DAB) staining was extracted from HA‐stained whole slide images. Each pixel in the 20× magnification HA‐extracted images had a squared resolution of 0.46 μm, so each MRI voxel corresponded to a 518 × 518 pixel region in the digital histology image. Each pixel in the HA‐extracted image was marked as ‘1’ if it is HA‐positive or ‘0’ if it is HA‐negative. The fraction of pixels occupied by HA was calculated within 518 × 518 pixel regions and that fraction represented a single voxel in the final MRI resolution‐matched image as previously described [22].

### 2.9 Statistical analyses

Statistical analyses were performed using prism 9 (GraphPad, San Diego, CA, USA), and data are presented as mean ± 1 standard error of the mean (SEM). Significant differences were identified using an unpaired Student's *t*‐test or a one‐way ANOVA with multiple comparisons with a significance level of 5%. A *q* value is shown in the case of multiple comparisons and is a *P*‐value which has been adjusted for the false discovery rate (FDR) using the two‐stage set up method by Benjamini, Krieger and Yekutieli. Correlations were evaluated using the Pearson correlation coefficient (*r*) with a significance level of 5%. Correlation matrices were created using prism 9.

The within‐subject test–retest coefficient of variation (CV_WS_) for T_1_, T_2_, MTR, ADC, R_2_*, G_d_ and G_l_ was calculated from repeated 4T1, 4T1/HAS3 and MDA‐MB‐231 LM2‐4 tumour MRI prior to and 24 h after saline using:

$${\mathrm{CV}}_{\mathrm{WS}}\;\left(\%\right)=100\times \sqrt{\frac{\Sigma {\left(\Delta /m\right)}^{2}}{2n}}$$

where *m* is the mean of all the pre‐saline and post‐saline MRI, Δ is the difference between the paired repeats, and *n* is the number of pairs [23].

## 3 Results

**Fig. 1:**
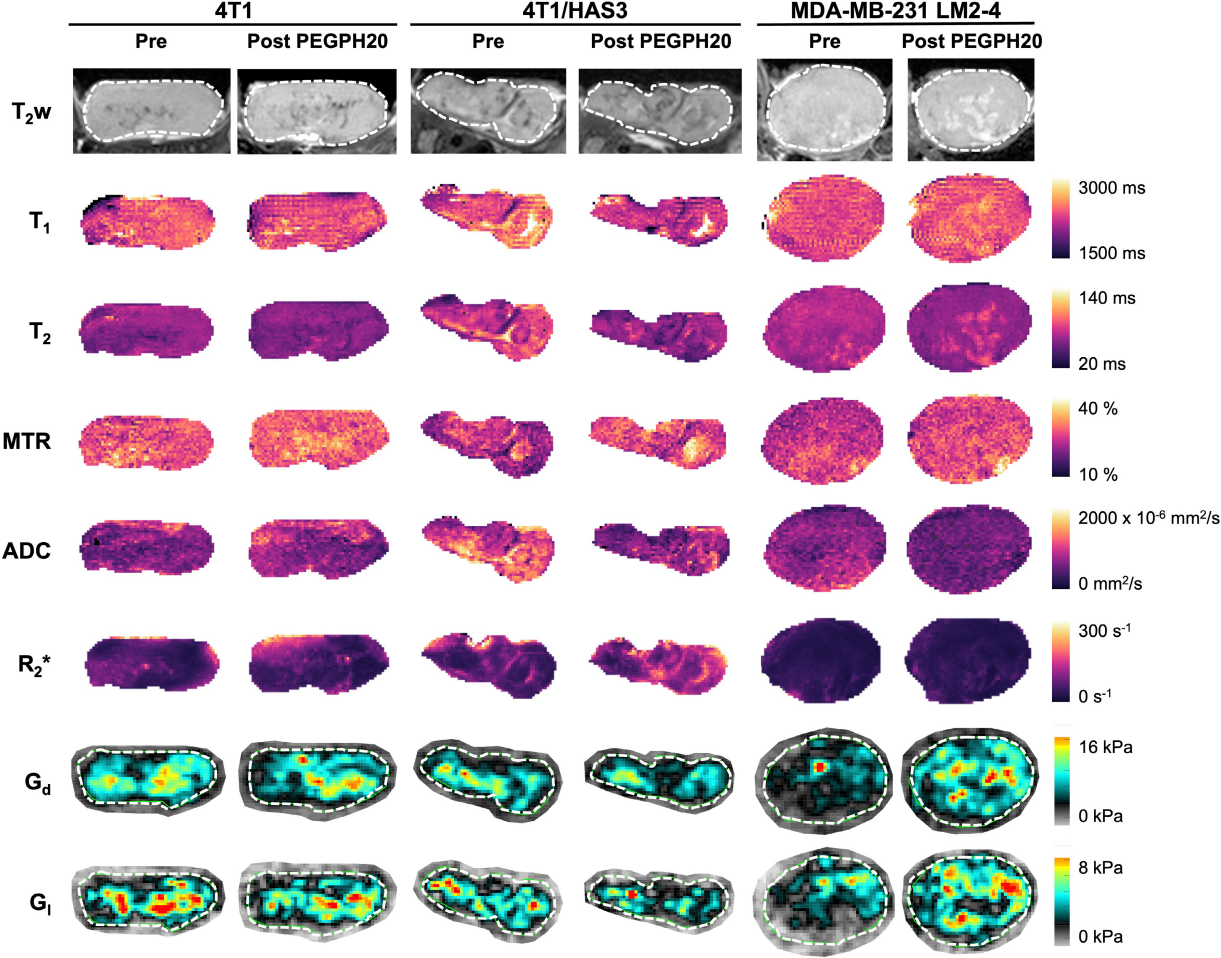
Multiparametric MRI before and after treatment with PEGPH20. Anatomical T_2_‐weighted (T_2_w) MRI and parametric maps of the longitudinal relaxation time (T_1_), transverse relaxation time (T_2_), magnetisation transfer ratio (MTR), apparent diffusion coefficient (ADC), transverse relaxation rate (R_2_*), elastic modulus (G_d_) and viscous modulus (G_l_) are shown for one representative 4T1, 4T1/HAS3 and MDA‐MB‐231 LM2‐4 tumour prior to and 24 h after PEGPH20 treatment (1 mg·kg^−1^). The whole tumour region of interest (ROI) is shown by a white dashed line where applicable.

Representative MRI data acquired from 4T1, 4T1/HAS3 and MDA‐MB‐231 LM2‐4 tumours treated with either PEGPH20 or saline are shown in Fig. 1 and Fig. S1, respectively; one tumour (pre‐ and post‐PEGPH20 or pre‐ and post‐saline) is shown per tumour model. The parametric maps of T_1_, T_2_, MTR, ADC, R_2_*, G_d_ and G_l_ revealed that PEGPH20‐induced homogeneous changes across viable tumour regions. The CV_WS_ (albeit, with a change in tumour volume), determined from the pre‐ and 24 h post‐saline data for all control tumours, were 2.2% (T_1_), 6.6% (T_2_), 5.0% (MTR), 16% (ADC), 16% (R_2_*), 13% (G_d_) and 16% (G_l_).

**Fig. 2:**
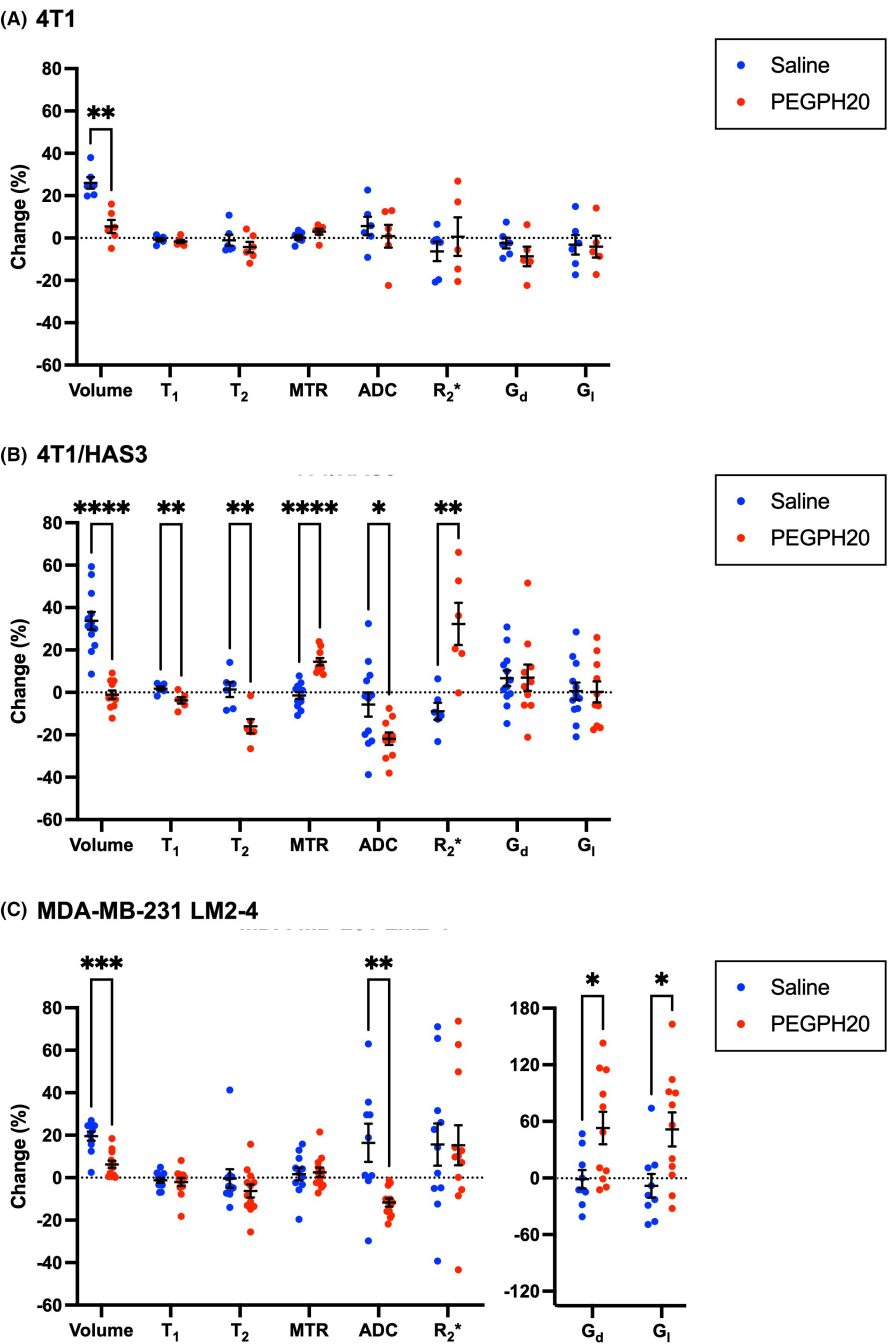
Changes in the quantitative volumetric and multiparametric MRI data following treatment with saline (blue) or PEGPH20 (red). Percentage change in tumour volume and MRI parameters between pre‐ and post‐treatment MRI for saline and PEGPH20‐treated mice bearing (A) 4T1, (B) 4T1/HAS3 or (C) MDA‐MB‐231 LM2‐4 tumours. PEGPH20 significantly reduced the growth of 4T1 (**q = 0.004), 4T1/HAS3 (****q < 0.0001) and MDA‐MB‐231 LM2‐4 (***q = 0.0005) tumours. PEGPH20 treatment did not significantly change the MRI biomarkers in 4T1 tumours (*q* > 0.05). PEGPH20 significantly reduced the longitudinal relaxation time (T_1_; **q = 0.01), transverse relaxation time (T_2_; **q = 0.006) and apparent diffusion coefficient (ADC; *q = 0.02), and significantly increased the magnetisation transfer ratio (MTR; ****q < 0.0001) and transverse relaxation rate (R_2_*; **q = 0.005) in 4T1/HAS3 tumours. PEGPH20 treatment of MDA‐MB‐231 LM2‐4 tumours significantly decreased ADC (**q = 0.007) and increased the elastic modulus (G_d_; *q = 0.03) and viscous modulus (G_l_; *q = 0.03). Data are the individual changes from median values of each tumour and the cohort mean ± 1 SEM. Sample sizes were: 4T1 saline *n* = 6 and PEGPH20 *n* = 6, 4T1/HAS3 saline *n* = 12 and PEGPH20 *n* = 12 (except for T_1_, T_2_ and R_2_* where saline *n* = 6 and PEGPH20 *n* = 6), and MDA‐MB‐231 LM2‐4 saline *n* = 11 and PEGPH20 *n* = 12. Multiple unpaired Student's *t*‐tests were conducted, and the resulting *q* value shown is the *P*‐value adjusted for the false discovery rate (FDR).

Mean values of MRI‐derived tumour volume, T_1_, T_2_, MTR, ADC, R_2_*, G_d_ and G_l_ are summarised for each tumour model before and following treatment with saline or PEGPH20 in Table S1. Percentage change values for all parameters following saline or PEGPH20 treatment are shown in Fig. 2.

PEGPH20 treatment significantly reduced the growth of 4T1, 4T1/HAS3 and MDA‐MB‐231 LM2‐4 orthotopic breast tumours (Table S1 and Fig. 2). 4T1/HAS3 tumours exhibited the fastest growth rate, followed by 4T1 tumours and MDA‐MB‐231 LM2‐4 tumours the slowest, with saline control tumours growing on average by 34%, 26% and 20%, respectively, over 24 h. PEGPH20 treatment did not significantly change any of the MRI biomarkers in 4T1 tumours. PEGPH20 treatment significantly increased MTR and R_2_*, and significantly reduced T_1_, T_2_ and ADC in 4T1/HAS3 tumours. PEGPH20 treatment of MDA‐MB‐231 LM2‐4 tumours significantly decreased ADC, and significantly increased tumour viscoelastic moduli G_d_ and G_l_. The relatively large increase in G_d_ and G_l_ following PEGPH20 (between 49% and 163%) occurred in six of the 11 MDA‐MB‐231 LM2‐4 tumours evaluated.

**Fig. 3:**
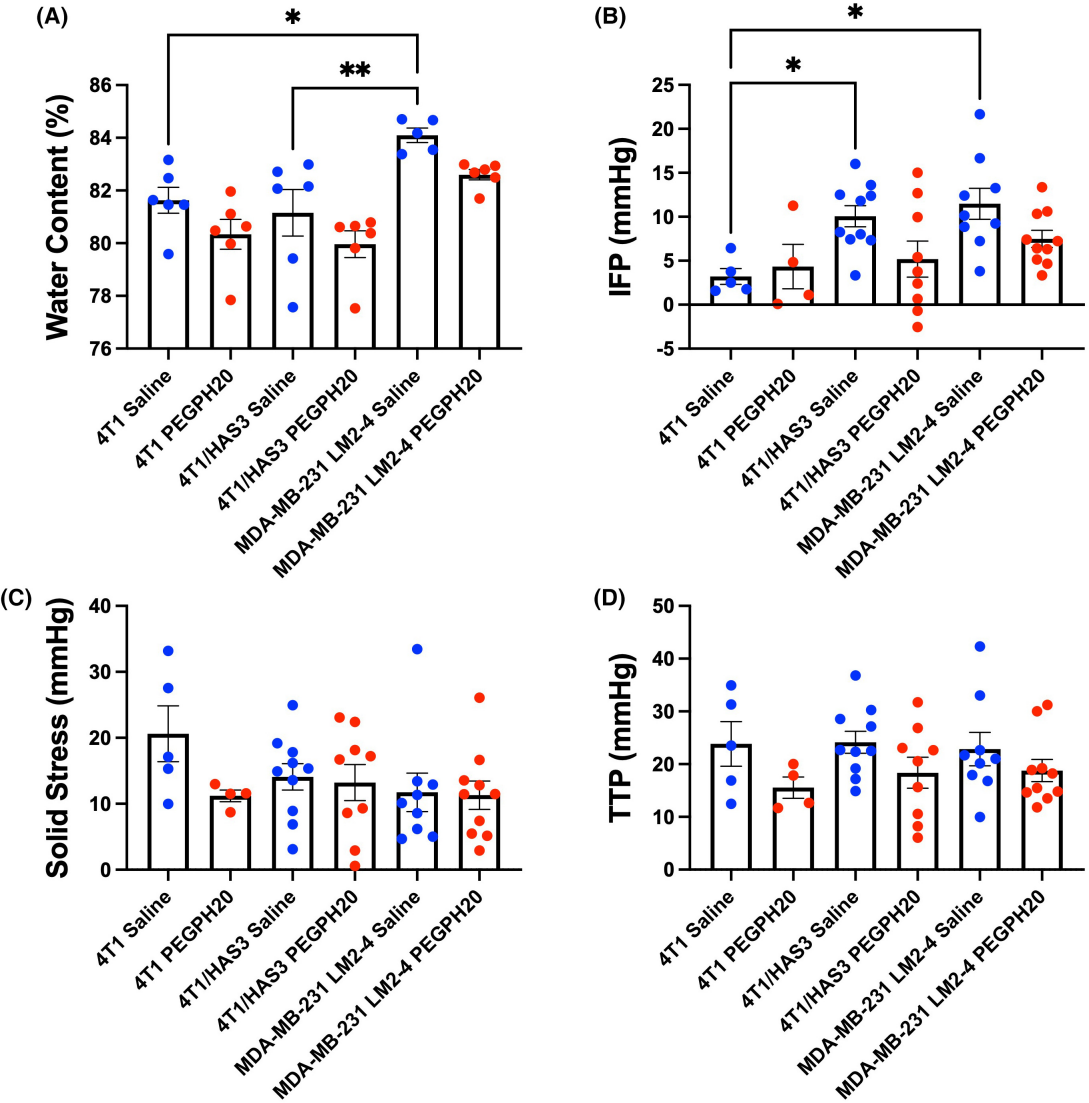
Differences between tumour models and the effect of PEGPH20 on tumour water content and pressure measurements. Tumour water content (A), interstitial fluid pressure (IFP) (B), solid stress (SS) (C) and total tissue pressure (TTP) (D) were measured from saline (blue) or PEGPH20 (red)‐treated 4T1, 4T1/HAS3 and MDA‐MB‐231 LM2‐4 tumours. MDA‐MB‐231 LM2‐4 tumours had higher water content than 4T1 (*P = 0.03, one‐way ANOVA with multiple comparisons) and 4T1/HAS3 tumours (**P = 0.006). Water content was measured *ex vivo* from one half of each tumour following post‐treatment MRI and tumour pressure measurements. IFP, SS and TTP were measured invasively *in vivo* following the post‐treatment MRI from one region within each tumour. 4T1 tumours had lower IFP compared to 4T1/HAS3 (*P = 0.05) and MDA‐MB‐231 LM2‐4 tumours (*P = 0.01). PEGPH20‐treated tumours did not have significantly different water content, IFP, SS, or TTP compared to saline controls in any of the three breast tumour models (ns; *P* > 0.05). Data are shown as one point for each tumour and are summarised by the cohort mean ± 1 SEM. For water content measurements: 4T1 saline *n* = 6 and PEGPH20 *n* = 6, 4T1/HAS3 saline *n* = 6 and PEGPH20 *n* = 6, and MDA‐MB‐231 LM2‐4 saline *n* = 5 and PEGPH20 *n* = 6. For IFP, SS and TTP measurements: 4T1 saline *n* = 5 and PEGPH20 *n* = 4, 4T1/HAS3 saline *n* = 10 and PEGPH20 *n* = 9, and MDA‐MB‐231 LM2‐4 saline *n* = 9 and PEGPH20 *n* = 10.

The MDA‐MB‐231 LM2‐4 tumours had a higher water content than the 4T1 (*P* = 0.03) and 4T1/HAS3 cohorts (*P* = 0.006; Fig. 3A). A lower water content was apparent in PEGPH20‐treated tumours compared to saline controls, but this was not statistically significant. 4T1 tumours had a lower IFP (3.2 mmHg) than 4T1/HAS3 (10.1 mmHg; *P* = 0.05) and MDA‐MB‐231 LM2‐4 tumours (11.5 mmHg; *P* = 0.01; Fig. 3B). The IFP, solid stress (SS) and total tissue pressure (TTP) of PEGPH20‐treated tumours was not significantly different to saline controls in any tumour model (Fig. 3B–D). A lower IFP was evident in the PEGPH20‐treated 4T1/HAS3 and MDA‐MB‐231 LM2‐4 cohorts, with the 4T1/HAS3‐treated tumours exhibiting a lower IFP (5.2 mmHg) compared to saline controls. MDA‐MB‐231 LM2‐4 saline control tumours had the highest IFP of all the tumour models and PEGPH20‐treated MDA‐MB‐231 LM2‐4 tumour IFP was markedly lower (7.5 mmHg).

**Fig. 4:**
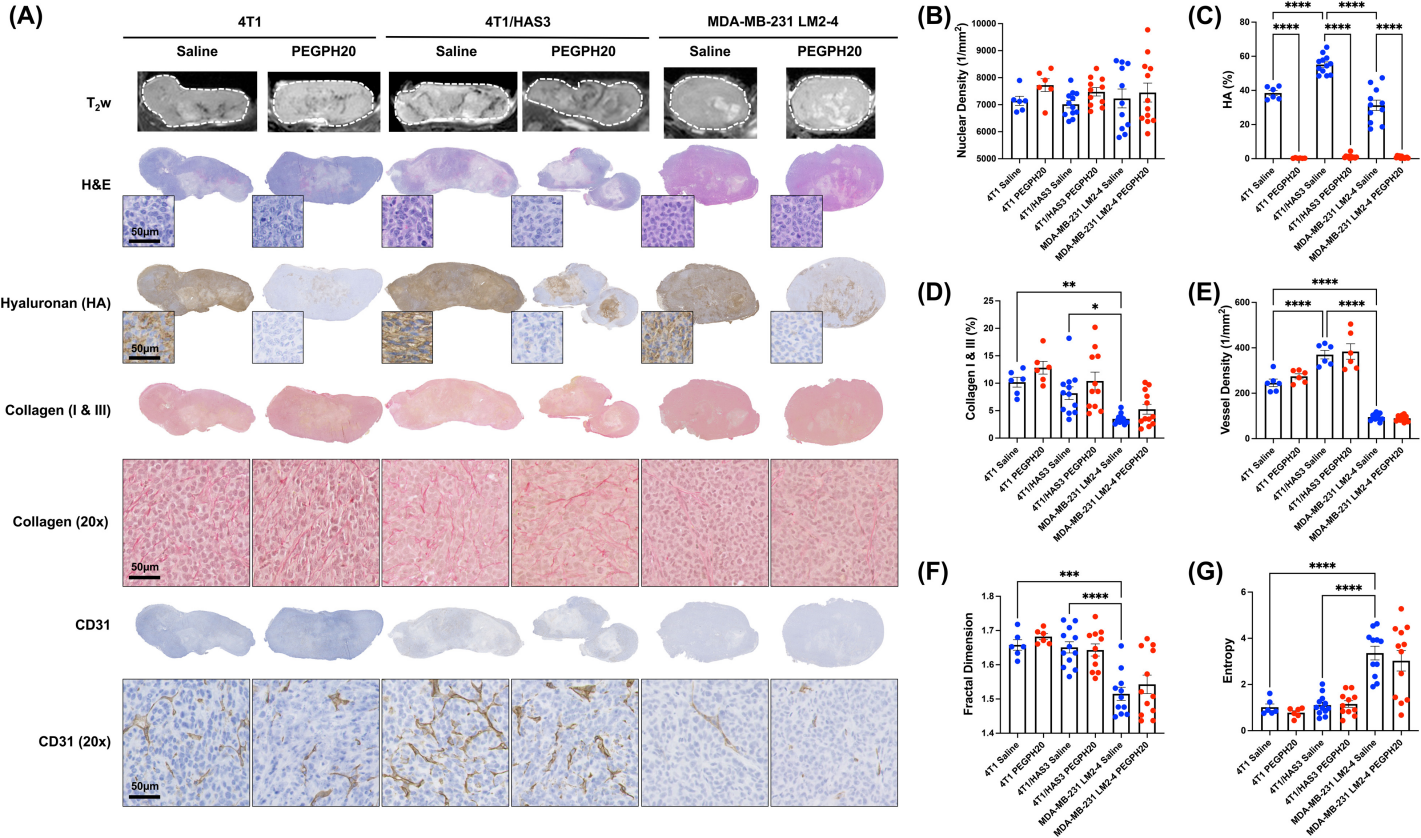
Quantitative digital pathology of MRI‐aligned tissue sections. (A) T_2_‐weighted (T_2_w) MRI of 4T1, 4T1/HAS3 and MDA‐MB‐231 LM2‐4 tumours, with histological images from aligned tissue sections taken from the same tumours stained with haematoxylin and eosin (H&E), HTI‐601 (hyaluronan/HA), picrosirius red (collagen I & III) or CD31 (blood vessels). Whole tumour section images and representative magnified images (20×) are shown for one saline control and one PEGPH20‐treated tumour per model. Scale bars shown are 50 μm. Histology sections were closely matched to the MRI. (B) Quantification of nuclear density from H&E staining. (C) Quantification of per cent HA from HTI‐601 staining revealed different baseline HA levels in 4T1, 4T1/HAS3 and MDA‐MB‐231 LM2‐4 tumours and a marked reduction in HA in all models with PEGPH20 treatment. (D) Quantification of per cent collagen I & III from picrosirius red staining showed lower collagen accumulation in MDA‐MB‐231 LM2‐4 tumours compared to 4T1 and 4T1 HAS3 tumours. (E) Blood vessel density quantified from CD31 staining showed that 4T1/HAS3 tumours had the most vasculature, followed by 4T1 tumours, and MDA‐MB‐231 LM2‐4 tumours had the least. Fractal dimension (F) and entropy (G) quantified from picrosirius red staining revealed that collagen fibre organisation in saline control MDA‐MB‐231 LM2‐4 tumours had a lower fractal dimension and entropy compared to saline control 4T1 and 4T1/HAS3 tumours. Data are shown as one point for each tumour and are summarised by the cohort mean ± 1 SEM. A one‐way ANOVA with multiple comparisons was used for each graph: *P < 0.05, **P < 0.01, ***P < 0.001 and ****P < 0.0001. Sample sizes were: 4T1 saline *n* = 6 and PEGPH20 *n* = 6, 4T1/HAS3 saline *n* = 12 and PEGPH20 *n* = 12 (except for vessel density where saline *n* = 6 and PEGPH20 *n* = 6), and MDA‐MB‐231 LM2‐4 saline *n* = 11 and PEGPH20 *n* = 12.

Good spatial correspondence was achieved between MRI and histology of imaging‐aligned tissue sections (Fig. S2). Representative post‐treatment anatomical T_2_‐weighted images and digitised images of aligned H&E, HTI‐601, picrosirius red and CD31 stained sections are shown in Fig. 4, alongside quantification of nuclear density (H&E), percent HA (HTI‐601), percent collagen I & III (picrosirius red), blood vessel density (CD31), collagen fractal dimension and collagen entropy. HTI‐601 staining of saline control tumours showed that 4T1/HAS3 tumours had the greatest HA accumulation (55%) compared to 4T1 (39%) and MDA‐MB‐231 LM2‐4 (31%) tumours. In all three models, HA accumulation was markedly and significantly lower in PEGPH20‐treated tumours (4T1 0.3%, 4T1/HAS3 1.2%, MDA‐MB‐231 LM2‐4 0.8%, all *P* < 0.0001). Saline control MDA‐MB‐231 LM2‐4 tumours contained significantly less collagen than 4T1 (*P* = 0.003) and 4T1/HAS3 (*P* = 0.02) tumours. Furthermore, the 2D distribution of collagen in saline control MDA‐MB‐231 LM2‐4 tumours had a significantly lower fractal dimension and higher entropy compared to saline control 4T1 (*P* = 0.0004) and 4T1/HAS3 (*P* < 0.0001) tumours. Greater collagen content was apparent in some PEGPH20‐treated tumours, particularly in the MDA‐MB‐231 LM2‐4 cohort, though this was not statistically significant overall. CD31 staining revealed that blood vessel density was highest in the 4T1/HAS3 saline control tumours (370 blood vessels per mm^2^ tissue), followed by 4T1 tumours (246 per mm^2^), and lowest in MDA‐MB‐231 LM2‐4 tumours (95 per mm^2^). PEGPH20 treatment did not elicit any significant effect on blood vessel density in any of the tumour models (*P* > 0.05).

**Fig. 5:**
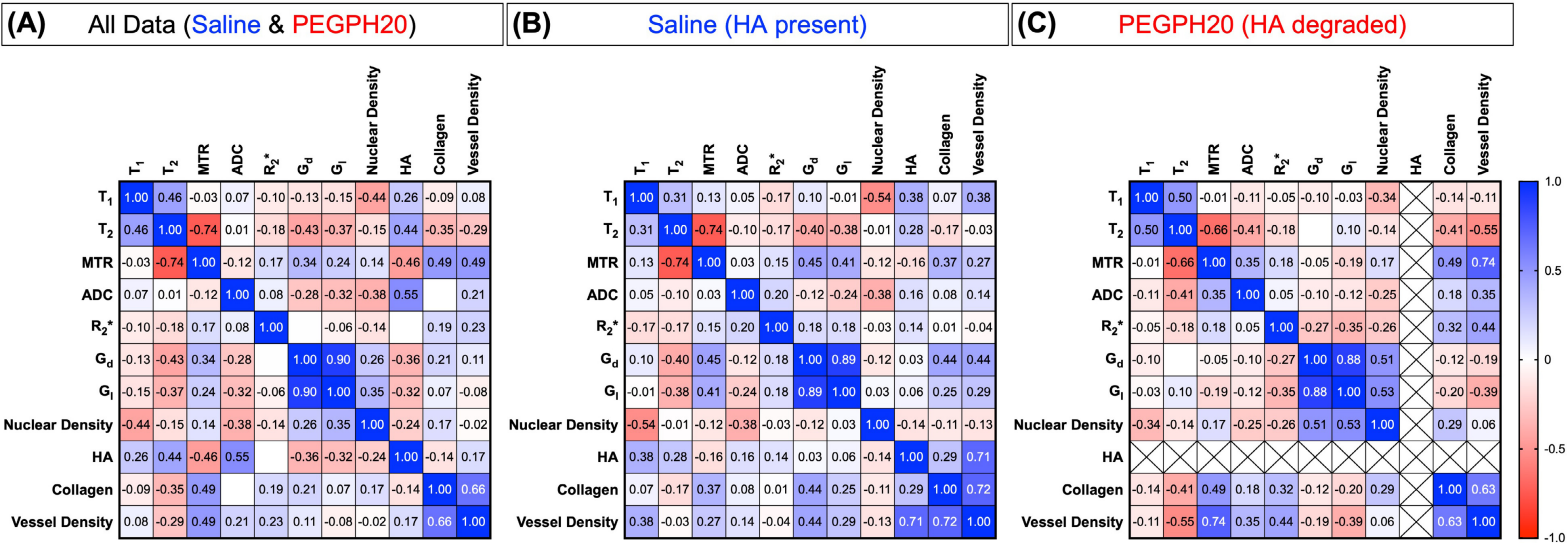
Correlation matrices of each MRI biomarker and histological marker. The correlation matrices show Pearson's correlation coefficient (*r*) for each pairing. Relationships were evaluated with all the data pooled together (A), and with saline (B) and PEGPH20‐treated (C) tumour data kept separate. MRI biomarkers were the longitudinal relaxation time (T_1_), transverse relaxation time (T_2_), magnetisation transfer ratio (MTR), apparent diffusion coefficient (ADC), transverse relaxation rate (R_2_*), elastic modulus (G_d_) and viscous modulus (G_l_). Nuclear density, percentage hyaluronan (HA), percentage collagen (I & III) and vessel density were calculated from H&E, HTI‐601, picrosirius red, and CD31 histological staining, respectively. Negligible levels of HA (< 2%) were detectable in the PEGPH20‐treated tumours; hence, per cent HA was excluded from the correlation analysis in this group (crossed‐out squares). For blank squares, −0.01 < *r* < 0.01. Sample sizes (*n*) and *P*‐values (*P*) for each correlation are shown in Fig. S3.

The clear spatial correspondence between the MRI and histology enabled an assessment of the relationships between the MRI biomarkers and the underlying pathology, both within tumours and between tumours. The between‐tumour correlation matrix determined for all the post‐treatment (saline & PEGPH20) measurements is shown in Fig. 5A. To further investigate the contribution of HA, the data were separated into saline control (Fig. 5B) and PEGPH20‐treated tumours (Fig. 5C). Sample sizes (*n*) and *P*‐values (*P*) for each correlation are shown in Fig. S3. T_2_ negatively correlated with MTR in all cases whether the saline and PEGPH20 data were evaluated together or kept separate (all data: *n* = 47, *r* = −0.74, *P* < 0.0001). T_1_ and ADC negatively correlated with nuclear density (all data T_1_: *n* = 47, *r* = −0.44, *P* = 0.002; all data ADC: *n* = 55, *r* = −0.38, *P* = 0.005).

**Fig. 6:**
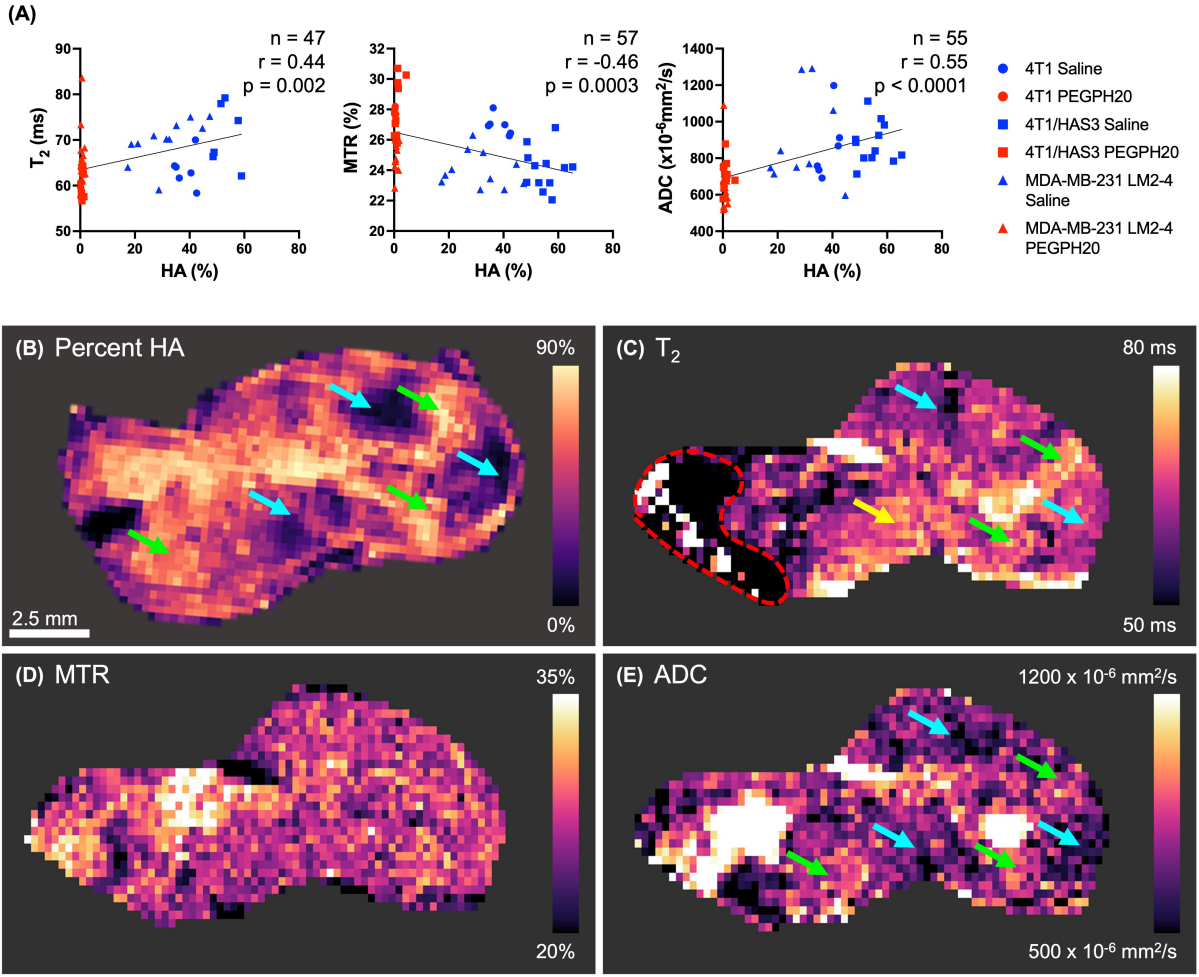
Inter‐ and intra‐tumour associations between MRI biomarkers and hyaluronan (HA). (A) Median values of MRI biomarkers were compared to percent HA (quantified from HTI‐601 staining) in saline (HA present; blue) and PEGPH20‐treated (HA degraded; red) 4T1, 4T1/HAS3, and MDA‐MB‐231 LM2‐4 breast tumours. Different shaped symbols indicate the different tumour models. The sample size (*n*), Pearson's correlation coefficient (*r*), and *P*‐value are shown by the linear regression they relate to. (B) An extracted parameter map from digital pathology of a whole tissue section stained for HA and processed to match the MRI resolution alongside aligned parametric maps of the (C) transverse relaxation time (T_2_), (D) magnetisation transfer ratio (MTR) and (E) apparent diffusion coefficient (ADC) for one saline control 4T1 tumour exhibiting intra‐tumour heterogeneity in HA accumulation. Green arrows highlight areas of high HA accumulation that corresponded to areas of high T_2_ and ADC. Blue arrows highlight regions of low HA related to areas of low T_2_ and ADC. Some regions of the T_2_ map appeared unrelated to HA accumulation (yellow arrow). MTR did not appear to have a spatial association with HA accumulation. The red dashed line indicates an artefact on the T_2_ map that was excluded from the analyses. The scale bar shown is 2.5 mm.

When analysing all the saline and PEGPH20‐treated data together, percent HA significantly correlated with T_2_, MTR and ADC (Figs 5A and 6). ADC exhibited the strongest, positive correlation with percent HA (all data: *n* = 55, *r* = 0.55, *P* < 0.0001). HA accumulation was positively correlated with T_2_ (all data: *n* = 47, *r* = 0.44, *P* = 0.002) and negatively correlated with MTR (all data: *n* = 57, *r* = −0.46, *P* = 0.0003). Although similar linear trendlines were apparent, there were no significant correlations between MRI biomarkers and percent HA when considering the saline data in isolation (Fig. 5B and Fig. S3B).

The saline control 4T1 tumours showed the greatest intra‐tumour heterogeneity in HA accumulation, and within this model, the relationships between T_2_, MTR, ADC and HA were spatially assessed using extracted parameter maps from digital pathology images of whole tumour sections processed to a pixel size that matched the MRI pixel size (Fig. 6B–E). Despite the numerical inter‐tumour association, areas of high HA accumulation were not visibly associated with low MTR and vice versa (Fig. 6D). There were some 4T1 tumour areas with high and low HA accumulation which aligned with high and low T_2_ (green and blue arrows, respectively, Fig. 6B,C). However, in other tumour regions, T_2_ and HA appeared unrelated (yellow arrow). As well as having the strongest numerical inter‐tumour association, visual comparison of ADC maps with HA staining images revealed that tumour regions with high ADC also had high HA accumulation and vice versa (all green and blue arrows; Fig. 6B,E).

**Fig. 7:**
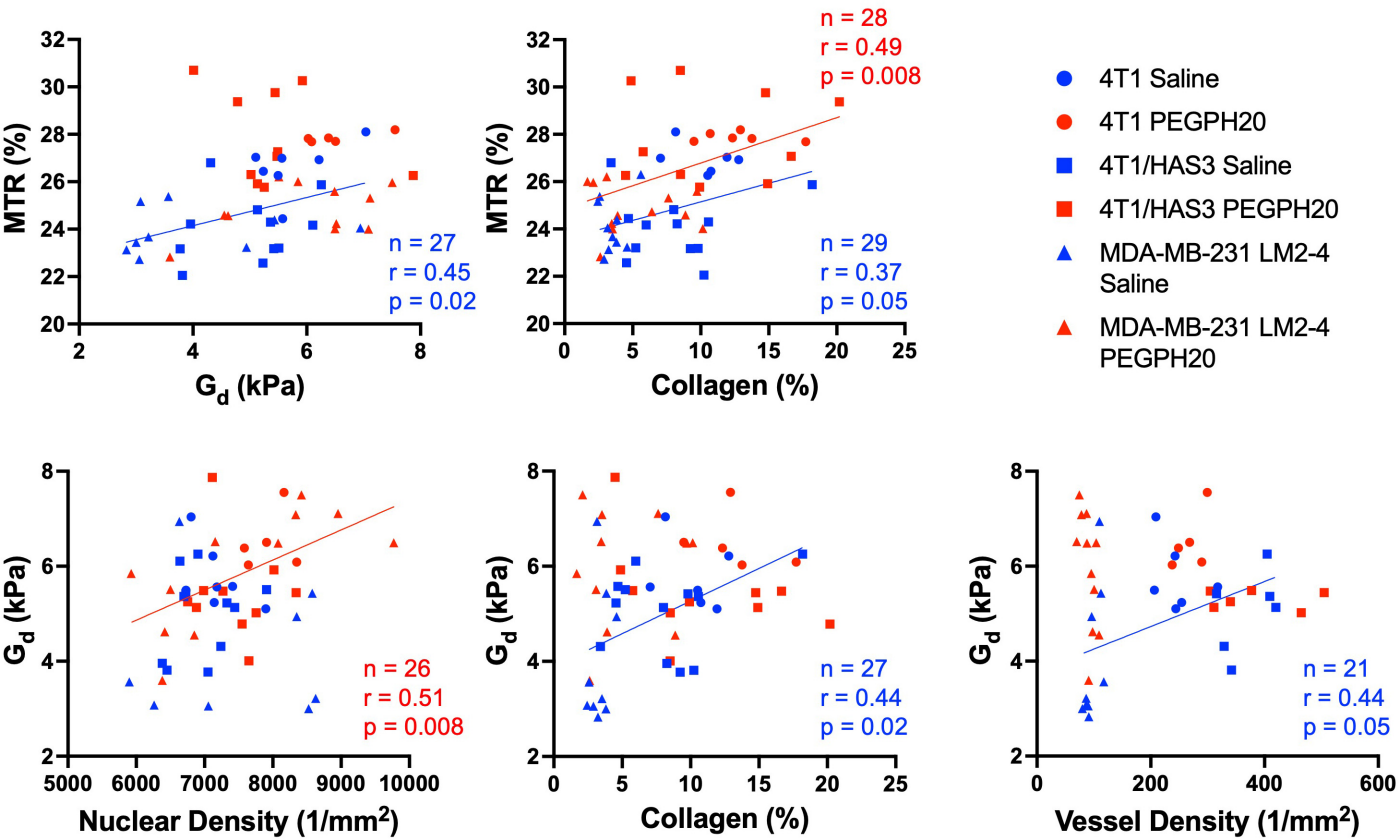
Relationships between the elastic modulus (G_d_), magnetisation transfer ratio (MTR) and histological markers can differ in saline (hyaluronan/HA present) and PEGPH20‐treated (HA degraded) tumours. Linear regression plots summarising the relationships between median G_d_, median MTR and quantitative histology are shown. Saline control and PEGPH20‐treated tumour data are shown in blue and red, respectively, with different shaped symbols indicating the three different breast tumour models. The sample size (*n*), Pearson's correlation coefficient (*r*), and *P*‐value are shown by the linear regression they relate to.

When HA was present in the tumour, MTR positively correlated with G_d_ (Fig. 7). However, in tumours where HA was degraded by PEGPH20, this relationship disappeared. MTR was found to positively correlate with collagen content in both saline and PEGPH20‐treated tumours. In saline control tumours, G_d_ positively correlated with collagen content and blood vessel density, but in the PEGPH20‐treated tumours, no such relationships were observed. Instead, G_d_ values measured in PEGPH20‐treated tumours positively correlated with nuclear density.

## 4 Discussion

Given the strong association of a stromal‐dense phenotype with tumour progression and impaired drug delivery, several strategies are being investigated to target key tumour ECM components, including HA, for therapeutic gain. The development of such stromal‐modulating therapies would be accelerated by imaging biomarkers that inform *in vivo* on changes in the ECM associated with therapeutic efficacy. This study assessed the ability of multiparametric MRI to map and quantify clinically translatable imaging biomarkers of the tumour microenvironment within breast tumour models with differing levels of HA accumulation and used these biomarkers to evaluate tumour response to PEGPH20 *in vivo*. The effective degradation of HA by PEGPH20 was histologically confirmed in MRI‐aligned tissue sections of all treated tumours.

Elevated HA accumulation was associated with a small growth advantage in the 4T1/HAS3 tumours compared to parental 4T1 tumours. PEGPH20 treatment delayed the growth of orthotopic 4T1, 4T1/HAS3 and MDA‐MB‐231 LM2‐4 breast tumours, consistent with previous reports in other preclinical models [7, 24, 25]. This growth delay was likely a consequence of collapse of the HA‐assembled gel–fluid matrix leading to a reduced extracellular space, breakdown of the previously HA‐bound tumour architecture, and, to a lesser extent given the short timepoint evaluated, biological effects driven by depletion of soluble growth factors in the extracellular space [7, 25].

The high avidity of HA for binding water, coupled with the reduction in water content following HA degradation, provide a compelling motivation to exploit multiparametric ^1^H MRI to investigate the role of HA in the tumour microenvironment and response to PEGPH20 *in vivo*. Quantitative mapping of the native T_1_ and T_2_ MRI relaxation times can inform on the distribution and extent of bound, structured or free tissue water molecules. MTR informs on the proportion of water bound to macromolecules and is sensitive to tumour ECM composition, as evidenced by correlations with fibrosis in preclinical models of pancreatic cancer, and collagen content in patient meningiomas [26, 27, 28]. The reduction in 4T1/HAS3 tumour T_1_ and T_2_ following treatment with PEGPH20 is consistent with a reorganisation of water molecules from the HA‐mediated gel‐fluid phase to a more bound state and/or loss of water from the tumour mass. A significant PEGPH20‐induced reduction in T_2_ has also been reported in a genetically engineered mouse (GEM) model of pancreatic ductal adenocarcinoma (PDAC) [29]. HA‐mediated fluid retention and expansion of the extracellular space with increasing HA accumulation may dilute the effects of MTR‐inducing ECM components such as collagen. Following HA degradation, their effects would no longer be attenuated, resulting in the increase in 4T1/HAS3 tumour MTR measured herein.

ADC is sensitive to the amount and mobility of water molecules and can inform upon various tissue properties, including the presence of macromolecules, cell membrane permeability, the characteristic sizes of water‐filled extracellular spaces and equilibrium of intracellular and extracellular water [30]. In viable tumour tissue, the movement of water is hindered by the high density of cells and extracellular matrix, resulting in restricted diffusion compared to normal tissues [31]. Following PEGPH20 treatment, ADC decreased in 4T1/HAS3 and MDA‐MB‐231 LM2‐4 tumours. This decrease in ADC is consistent with both an overall decrease in tumour water content and a reduction in the size of the extracellular space following degradation of HA, thereby bringing tumour components that restrict water diffusion closer together (e.g. blood vessels, cells and collagen). It was encouraging to observe this response in the MDA‐MB‐231 LM2‐4 cohort, one of the tumour models with lower HA accumulation. The 4T1 tumours had a lower IFP compared to 4T1/HAS3 and MDA‐MB‐231 LM2‐4 tumours, suggesting that HA polymers in this tumour model bound and coordinated fewer water molecules. Visual assessment of MRI‐aligned 4T1 tissue sections shows that ADC is associated with HA accumulation but, given the lower IFP, treatment with PEGPH20 may only elicit a small change in water content and mobility insufficient to significantly change ADC in this model. These data highlight the potential of ADC as a biomarker of HA and its therapeutic degradation in stromal‐dense tumours with elevated IFP.

IS‐MRI revealed that 4T1/HAS3 tumours exhibited a faster baseline R_2_* than 4T1 and MDA‐MB‐231 LM2‐4 tumours, suggesting a more vascularised phenotype, and supported by their relatively high vascular density quantified from CD31 immunohistochemistry. Accordingly, treatment with PEGPH20 increased R_2_* in the 4T1/HAS3 cohort, consistent with decompression of blood vessels and increased accessibility of deoxygenated (paramagnetic) erythrocytes within the perfused tumour vasculature. Quantitation of tumour R_2_* as a proxy for blood vessel density may provide a predictive biomarker of PEGPH20 response. Perfusion MRI techniques utilising exogenously administered paramagnetic contrast agents may provide more sensitive approaches to inform on tumour vascular changes following HA degradation. Indeed PEGPH20‐mediated improvements in tumour perfusion/permeability and fractional blood volume have been reported in models of PDAC using dynamic contrast enhanced MRI and susceptibility contrast MRI [32, 33].

There is a strong rationale for incorporating MRE into multiparametric MRI investigations of stromal‐dense cancers to assess the underlying biology and response to drugs that target the ECM. MRE revealed that 4T1/HAS3 tumours had lower G_d_ and G_l_ compared to 4T1 tumours which, coupled with quantitation of HTI‐601 staining, suggests that greater HA accumulation contributes to a lowering of the elastic and viscous moduli. Despite this, HA degradation by PEGPH20 induced no change in viscoelastic moduli of 4T1 and 4T1/HAS3 tumours. It may be that HA accumulation during tumour development is associated with a softer and less viscous phenotype, but the extent of HA itself does not directly contribute to tumour viscoelastic properties. Interestingly, PEGPH20 induced an unprecedented ∼ 80% increase in G_d_ and G_l_ in MDA‐MB‐231 LM2‐4 tumours. Using MRE and digital pathology, we have previously shown that collagen is a major determinant of the elevated tumour elasticity and viscosity in MDA‐MB‐231 LM2‐4 tumours [18]. Herein, quantitative histology suggested a higher collagen content, with a more homogeneous and dense distribution (lower entropy and higher fractal dimension) in three of the six MDA‐MB‐231 LM2‐4 tumours that exhibited an increase in G_d_ and G_l_ following administration of PEGPH20. Accumulation of HA may diminish the contribution of collagen to ECM stiffening in these tumours, which is abrogated by PEGPH20, hence the marked increase in tumour stiffness measured by MRE.

Despite promising preliminary data, the phase III clinical trial of PEGPH20 in combination with standard‐of‐care chemotherapy (HALO‐109‐301) failed to improve progression free or overall survival of patients with previously untreated HA ‘high’ metastatic PDAC [34, 35]. The reasons for the failure of the trial are unclear. Whilst stromal modulatory strategies such as PEGPH20 are being developed to enhance drug delivery and hence treatment, there is also the potential to promote tumour progression by removing barriers to invasion and metastasis [36, 37, 38]. It is interesting to speculate that translation of the PEGPH20‐induced increase in tumour stiffness seen in the MDA‐MB‐231 LM2‐4 tumours to a patient cohort may be indicative of poor prognosis. Future work should evaluate whether PEGPH20 treatment can elicit a detrimental effect with respect to tumour progression and assess how this would translate to patients treated with ECM‐targeted therapies. Several oncological MRE studies have typically reported tumour softening associated with cell death following effective treatment [17, 39]. The data herein demonstrate the ability of MRE to measure pharmacologically induced *increases* in tumour stiffness and support further evaluation of G_d_ and G_l_ as binary imaging biomarkers of treatment response. Furthermore, G_d_ and G_l_ derived from 3D MRE measurements were highly correlated and changed in a very similar way following PEGPH20 treatment. Therefore, if MRE were to be used to evaluate human tumour response to PEGPH20, a single measure of stiffness derived from 2D MRE may likely be sufficient and enhance clinical translation of the method.

A dense ECM can increase solid stress, elevate IFP, impede drug delivery and promote breast tumour progression and metastasis [1, 3, 40]. Previous studies have reported that degradation of HA with PEGPH20 can reduce IFP and suggest this is associated with improved drug delivery and tumour response to therapy [7, 9, 11, 12, 13]. In our study, there was a non‐significant trend of lower IFP in PEGPH20‐treated 4T1/HAS3 and MDA‐MB‐231 LM2‐4 tumours compared to saline controls. PEGPH20‐treated tumours had lower water content compared to saline control tumours, although this was not statistically significant. Previous studies have shown that whilst tumour IFP and blood vessel lumen area return to pre‐treatment values relatively quickly (∼ 48 h) following PEGPH20 treatment *in vivo*, the reduction in tumour water content is sustained for at least 120 h [7]. The relative longevity of this response should thus provide a useful time window in which to probe the characteristics of tumour water with MRI when evaluating PEGPH20 or other interventions that degrade HA in the clinic.

Correlation matrices were used to provide a deeper assessment of the relationship of the MRI biomarkers to the underlying histology in the presence and absence of HA. The MRI biomarkers investigated may be sensitive, but not specific to HA. To interrogate their sensitivity to HA, PEGPH20‐treated data were included with the intention to provide ‘low HA’ values. This approach reduced the influence of other tumour characteristics to each correlation, as well as increasing the dynamic range of HA values available. ADC exhibited the strongest positive correlation with per cent HA, reaffirming ADC as the most sensitive biomarker of tumour response to PEGPH20, most likely due to its sensitivity to the reduction in size of the extracellular space and quantity of water molecules able to diffuse. PEGPH20‐induced reductions in ADC of similar magnitude were recently reported from early phase clinical trial data in patients bearing tumours from other anatomical sites including colon, lung and pancreas [41].

The positive correlation between ADC and cellularity has been previously reported in several tumour types, including breast cancer [42, 43]. Increased cell density restricts water diffusion, thereby decreasing ADC. Interestingly, ADC did not significantly correlate with nuclear density in the PEGPH20‐treated tumours. This highlights that ADC is not always a biomarker of cellularity and can be influenced by other tumour components [43]. HA degradation leads to a reduction of the extracellular space bringing many tumour constituents (e.g. cells, stroma, blood vessels etc) closer together, which will restrict the movement of water in a way not only related to tumour cellularity.

T_2_ and MTR are other, less specific parameters related to percent HA. Greater HA accumulation results in elevated fluid retention, as well as extracellular expansion, which together regulate the amount of free water, thus increasing T_2_ and attenuating the effects of MT‐inducing components of the ECM such as fibrillar collagen.

**Fig. 8:**
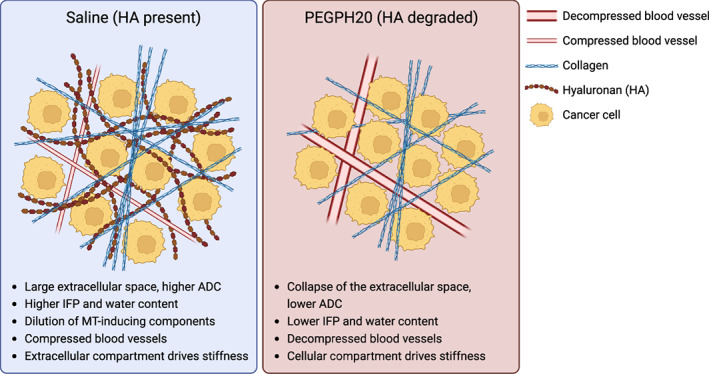
A simplified graphic summarising the hypothesised explanation for the different characteristics of preclinical breast tumours treated with either saline (blue) or PEGPH20 (red). ADC, apparent diffusion coefficient; ECM, extracellular matrix; HA, hyaluronan; and IFP, interstitial fluid pressure. Created with BioRender.com.

The correlation matrices suggest that HA degradation alters the tumour microenvironment such that MTR and G_d_ are no longer sensitive to the same tumour microenvironmental components. Whilst HA degradation increases MTR, as shown by an upward shift in the trendline of the PEGPH20 treated tumours, it does not affect the sensitivity of MTR to collagen deposition. In saline control tumours, G_d_ positively correlated with collagen content and blood vessel density, reaffirming them as determinants of MRE‐derived tumour elastic properties [18, 44]. However, in the PEGPH20‐treated tumours, these relationships disappeared, with G_d_ positively correlated with nuclear density instead. These associations suggest that HA degradation affects the integrity of the ECM, reducing the ability of fibrillar collagen and blood vessels to increase tumour stiffness. As a result, the viscoelastic properties of PEGPH20‐treated tumours become more reliant on the cellular network. A summary graphic describing the influence of HA on the breast tumour microenvironment based on these relationships is shown in Fig. 8.

## 5 Conclusions

Multiparametric MRI has the potential to non‐invasively inform on prognosis and improve risk stratification for patients with stromal‐dense cancer. Numerous stromal modulating strategies that target the biophysical properties of tumours to improve cancer therapies are being exploited, whose clinical development would benefit from imaging biomarkers that accurately inform on tumour response. The data herein show that ADC is a sensitive biomarker of HA modulation, providing a strong rationale to incorporate DWI to accelerate the development of ECM‐targeted therapies. The study also highlights the potential of MRE to inform on tissue mechanics and enable expedient adaptation of treatment regimes. Whilst the development of PEGPH20 has been halted, other approaches to degrade HA in cancer are being investigated [45, 46, 47, 48, 49], for which imaging‐embedded trials would benefit from inclusion of DWI and elastography.

## Conflict of interest

David Kang is an employee of Halozyme Therapeutics. Barbara Blouw was an employee of Halozyme Therapeutics at the time of the study. David Kang and Barbara Blouw are shareholders of Halozyme Therapeutics.

## Author contributions

ELR, JCB, YJ, and SPR were involved conceptualization. JL, JCB, and SPR were involved in supervision. ELR, JL, KZ‐P, JKRB, JS, CC, BB, DK, RS, YJ, and SPR were involved in methodology and resources. ELR and JL were involved in data acquisition. ELR and KZ‐P were involved in data analysis. ELR was responsible for manuscript preparation (initial draft). ELR, JL, KZ‐P, JKRB, JS, CC, BB, DK, RS, JCB, YJ, and SPR were involved in manuscript review.

### Peer review

The peer review history for this article is available at https://www.webofscience.com/api/gateway/wos/peer‐review/10.1002/1878‐0261.13437.

## Supporting information


**Fig. S1.** Multiparametric MRI before and 24‐h after saline.
**Table S1.** Summary of the quantitative volumetric and multiparametric MRI data determined prior to and post‐treatment.
**Fig. S2.** Representative MRI and aligned histology images.
**Fig. S3.** Sample sizes (blue) and *P*‐values (red) for the correlation matrices of each MRI biomarker and histological marker shown in Fig. 5.

## Data Availability

The data generated in this study are available upon reasonable request from the corresponding author.
