# Supplementary material for: Investigating the contribution of hyaluronan to the breast tumour microenvironment using multiparametric MRI and MR elastography
Source: Mol Oncol. 2023 May 3;17(6):1076–92. doi: 10.1002/1878-0261.13437 (PMC10257424; doi:10.1002/1878-0261.13437)
Supplement: Supplementary file 1 — Fig. S1. Multiparametric MRI before and 24‐h after saline. Table S1. Summary of the quantitative volumetric and multiparametric MRI data determined prior to and post‐treatment. Fig. S2. Representative MRI and aligned histology images. Fig. S3. Sample sizes (blue) and P‐values (red) for the correlation matrices of each MRI biomarker and histological marker shown in Fig. 5. [file MOL2-17-1076-s001.docx]

Supporting Information:


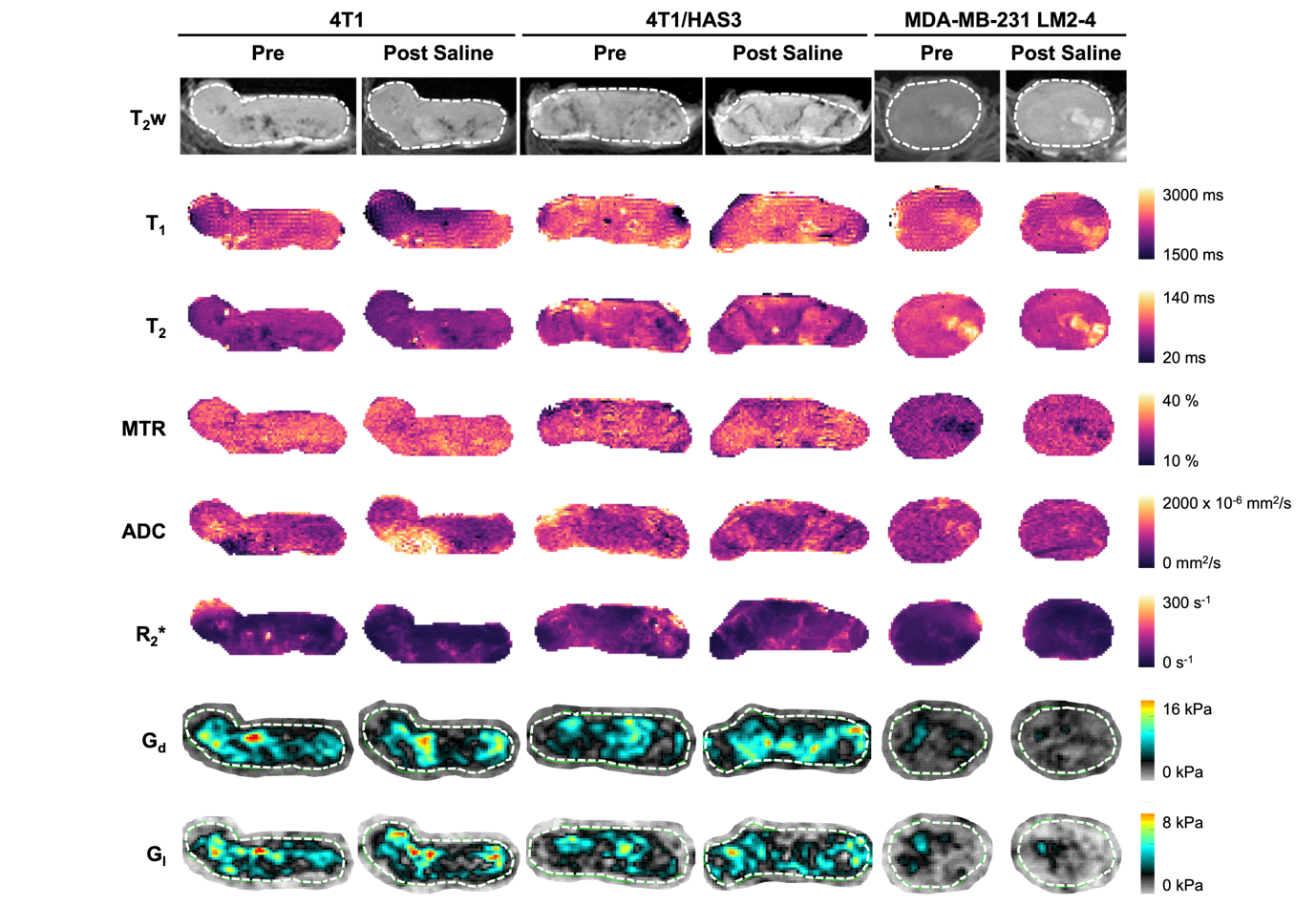


**Figure S1: Multiparametric MRI before and 24-hours after saline.** Anatomical T_2_-weighted (T_2_w) MRI and parametric maps of the longitudinal relaxation time (T_1_), transverse relaxation time (T_2_), magnetisation transfer ratio (MTR), apparent diffusion coefficient (ADC), transverse relaxation rate (R_2_*), elastic modulus (G_d_) and viscous modulus (G_l_) for a representative 4T1, 4T1/HAS3 and MDA-MB-231 LM2-4 tumour prior to and 24 hours after treatment with saline. The whole tumour region of interest (ROI) is shown by a white dashed line where applicable.

**Table S1: Summary of the quantitative volumetric and multiparametric MRI data determined prior to and post-treatment.** Mean MRI-derived tumour volume, longitudinal relaxation time (T_1_), transverse relaxation time (T_2_), magnetisation transfer ratio (MTR), apparent diffusion coefficient (ADC), transverse relaxation rate (R_2_*), elastic modulus (G_d_) and viscous modulus (G_l_) for 4T1, 4T1/HAS3 and MDA-MB-231 LM2-4 tumours pre-saline, post-saline, pre-PEGPH20 and post-PEGPH20. The mean percentage change is also shown, with percentage change values in bold indicating a significant difference between saline controls and PEGPH20 treated tumours (q<0.05; p-value from multiple unpaired Student’s t-tests adjusted for the false discovery rate). Data are shown as mean ± 1 SEM.


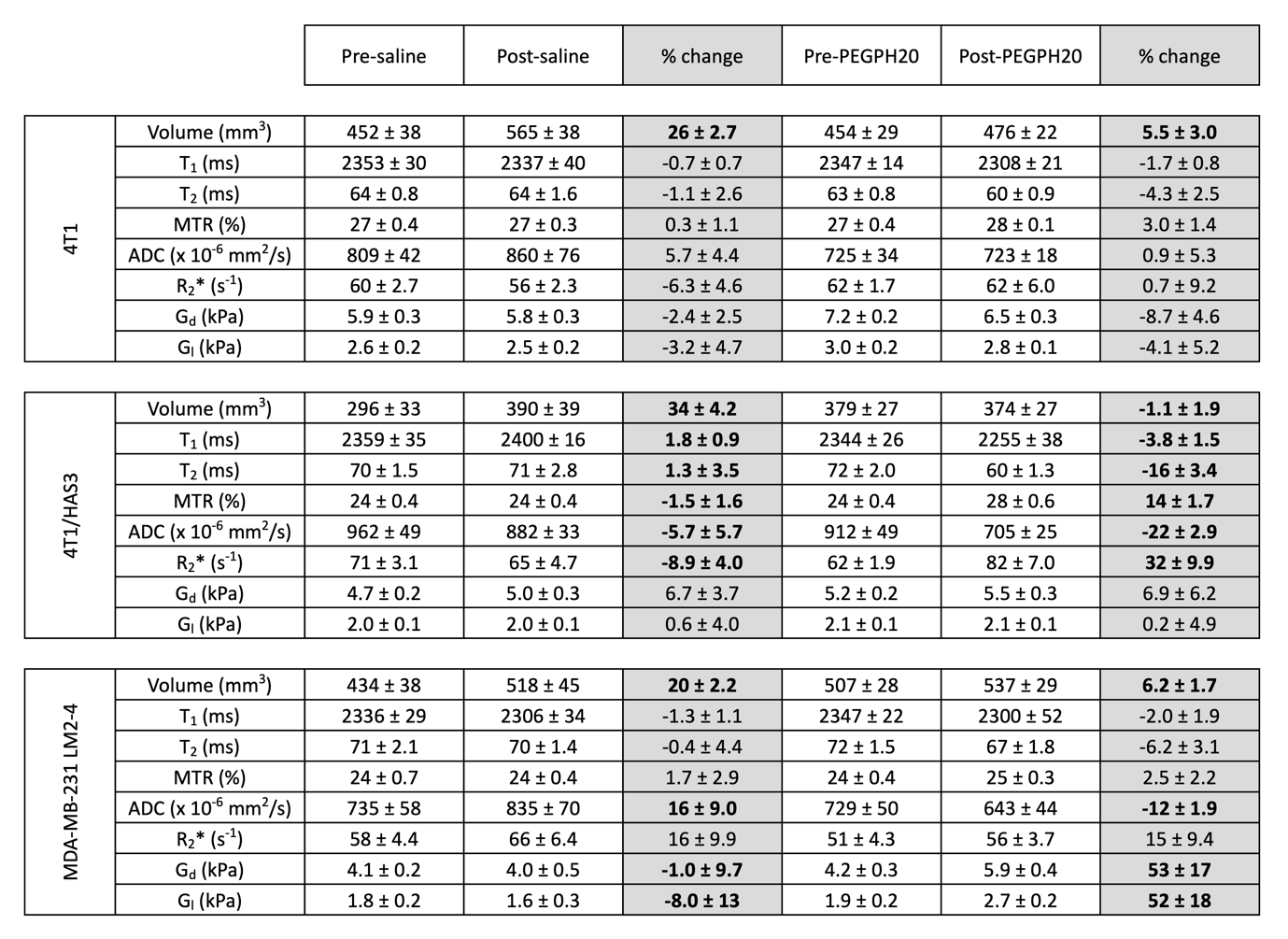


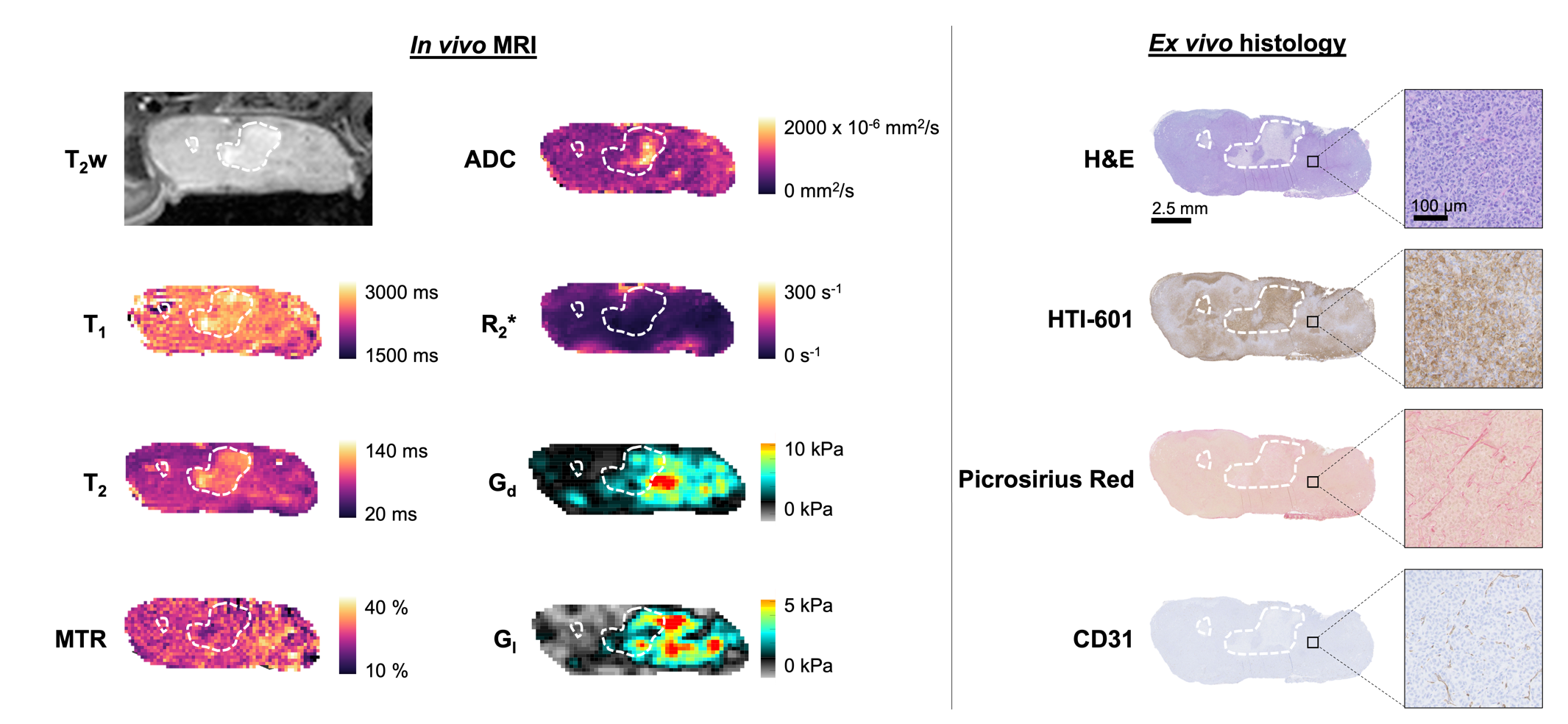


**Figure S2: Representative MRI and aligned histology images.** Histology images are from whole tissue sections (scale bar 2.5 mm), with 10x magnification snapshots also shown (scale bar 100 μm). The MRI and histology are from a representative MDA-MB-231 LM2-4 tumour following treatment with saline. MRI included anatomical T_2_-weighted (T_2_w) MRI and parametric maps of the longitudinal relaxation time (T_1_), transverse relaxation time (T_2_), magnetisation transfer ratio (MTR), apparent diffusion coefficient (ADC), transverse relaxation rate (R_2_*), elastic modulus (G_d_) and viscous modulus (G_l_). Areas of necrosis are delineated by a white dashed line and were excluded from the quantitative analyses. MRI-aligned tissue sections were stained using haematoxylin and eosin (H&E), HTI-601 (hyaluronan/HA), picrosirius red (collagen I & III), and CD31 (blood vessels).


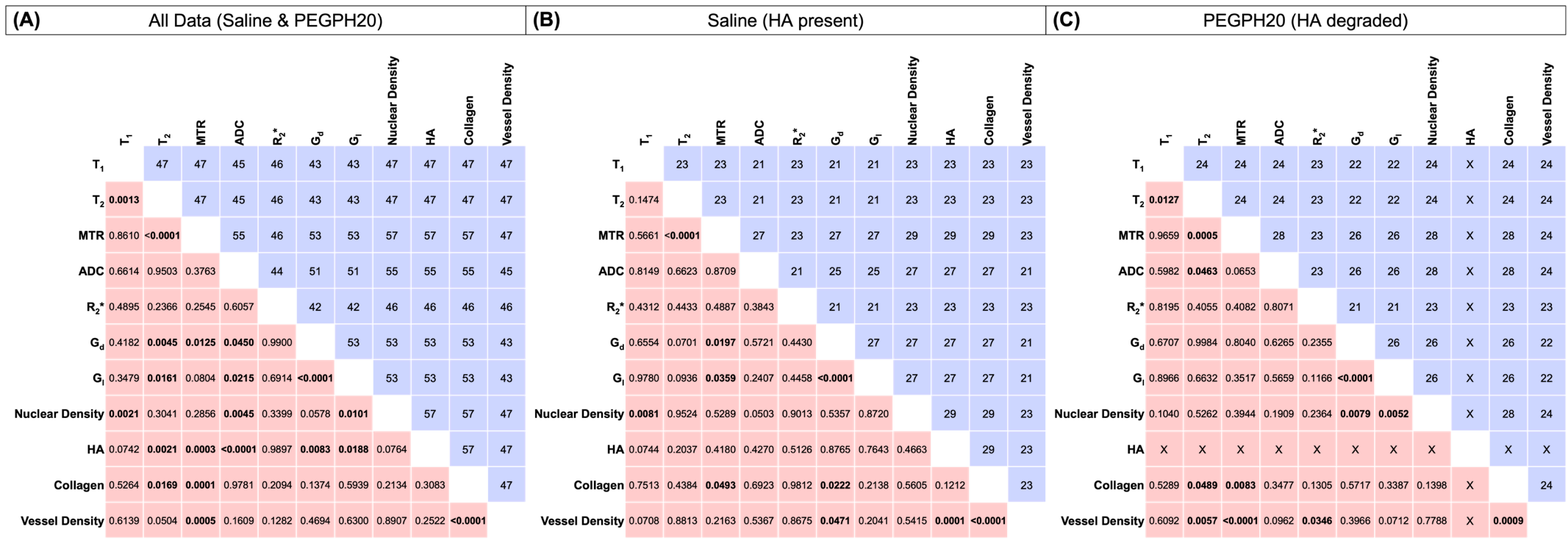


**Figure S3: Sample sizes (blue) and p-values (red) for the correlation matrices of each MRI biomarker and histological marker shown in Figure 5.** Relationships were evaluated with all the data pooled together **(A)**, and with saline **(B)** and PEGPH20 treated **(C)** tumour data kept separate. Significant p-values are shown in bold (p<0.05). MRI included anatomical T_2_-weighted (T_2_w) MRI and parametric maps of the longitudinal relaxation time (T_1_), transverse relaxation time (T_2_), magnetisation transfer ratio (MTR), apparent diffusion coefficient (ADC), transverse relaxation rate (R_2_*), elastic modulus (G_d_) and viscous modulus (G_l_). Nuclear density was quantified from haematoxylin and eosin (H&E) staining, percent hyaluronan (HA) from HTI-601 staining, percent collagen (I & III) from picrosirius red staining, and blood vessel density from CD31 staining.
